# Grazing intensity shapes soil fungal diversity and co-occurrence networks across soil depths

**DOI:** 10.3389/fmicb.2026.1941617

**Published:** 2026-09-30

**Authors:** Jincheng Shi, Yao Su, Fei Wang, Qianju Wang, Jianhao Yu, Jing Shi, Xiang Zhao, Kuanhu Dong, Jia Mi

**Affiliations:** 1 Shanxi Key Laboratory of Ecological Restoration on Loess Plateau, Institute of Loess Plateau, Shanxi University, Taiyuan, China; 2 College of Environment and Resources Sciences, Shanxi University, Taiyuan, China; 3 College of Grassland Science, Shanxi Agricultural University, Taigu, China; 4 Observation and Research Station for Grassland Ecosystem in the Loess Plateau, Youyu, China; 5 Field Scientific Observation and Research Station of the Ministry of Education for Subalpine Grassland Ecosystem in Shanxi, Ningwu, China

**Keywords:** agro-pastoral ecotone, fungal co-occurrence network, grazing intensity, high-throughput sequencing, soil fungal diversity, vertical soil profile

## Abstract

Grazing is an important disturbance affecting grassland ecosystems in agro-pastoral ecotones, yet its effects on soil fungal diversity and co-occurrence networks across vertical soil profiles remain unclear. We selected a typical grassland at the junction of the Loess Plateau and the Mongolian Plateau in northern China and established four grazing intensities: ungrazed control (CK), light grazing (LG), moderate grazing (MG), and heavy grazing (HG). Soil samples were collected from three layers (0–2, 2–10, and 10–20 cm). Soil physicochemical properties, high-throughput sequencing, co-occurrence network analysis, and structural equation modeling (SEM) were used to examine fungal community responses and their potential associated pathways. Grazing intensity and soil depth significantly influenced soil physicochemical properties and fungal community composition. Fungal α-diversity exhibited soil layer-dependent responses to grazing intensity, with light and moderate grazing maintaining higher fungal richness in surface soils than heavy grazing, whereas fungal richness responses differed among deeper soil layers. Factorial PERMANOVA showed significant effects of grazing intensity (*R*^2^ = 0.145, *P* = 0.043) and soil depth (*R*^2^ = 0.151, *P* < 0.001) on fungal community composition, whereas their interaction was not significant. Co-occurrence network analysis revealed variation in fungal network organization among grazing intensities, with moderate grazing associated with higher network connectivity and heavy grazing associated with higher network modularity. Piecewise structural equation modeling further indicated indirect associations between grazing intensity and fungal diversity involving vegetation biomass, soil resources, soil pH, and microbial biomass. Overall, our findings demonstrate that grazing effects on soil fungal communities are strongly soil layer-dependent and are shaped by interactions among plant inputs, soil environmental conditions, and microbial processes. This study provides insights into the ecological consequences of grazing intensity and the mechanisms underlying fungal community responses in agro-pastoral ecotone grasslands.

## 1 Introduction

The agro-pastoral ecotone is an important ecological transition zone in northern China. This region is jointly influenced by agricultural activities and grazing, and its ecological environment is relatively fragile. Long-term inappropriate grazing has led to grassland degradation, mainly manifested as declines in vegetation productivity and the deterioration of soil ecosystem functions. Previous studies have shown that grazing, as a major anthropogenic disturbance, can substantially alter grassland ecological processes through livestock foraging, trampling, and excreta deposition, thereby exerting profound effects on soil physicochemical properties and biological communities (Chen et al., 2021a; Wang et al., 2022c; Tu et al., 2025). In recent years, although grazing exclusion and grazing restriction have been implemented in China to improve vegetation conditions, their effects on soil microbial communities remain controversial, and no consensus has yet been reached (Chen et al., 2020; Shu et al., 2024). Therefore, investigating the effects of different grazing intensities on soil microbial communities in agro-pastoral ecotone is of considerable ecological significance.

Soil microorganisms are important components of grassland ecosystems because they play a central role in driving nutrient cycling and maintaining ecosystem stability (Zhang et al., 2017; Ding et al., 2023). Among soil microorganisms, fungi are major decomposers that contribute to the degradation of recalcitrant organic matter and facilitate plant nutrient acquisition (Song et al., 2022; Manici et al., 2024). We focused specifically on fungi because their close involvement in plant-derived carbon turnover and their sensitivity to grazing-induced changes in vegetation inputs, soil structure, and resource availability make them an ecologically informative group for assessing belowground responses to grazing. Consequently, changes in fungal community composition may have important consequences for ecosystem nutrient cycling (Klumpp et al., 2009; Chen et al., 2020). Numerous studies have shown that grazing can reshape the structure and function of soil microbial communities by altering plant community composition and soil physicochemical properties (Chen et al., 2021a; Wang et al., 2022c; Xu et al., 2023). Grazing regulates fungal communities through multiple direct and indirect pathways. On the one hand, livestock foraging and excreta deposition alter the input patterns and spatial distribution of nutrients such as carbon and nitrogen. On the other hand, trampling changes soil bulk density, pore structure, and moisture conditions, thereby restructuring microbial habitat conditions. In addition, grazing-induced changes in plant community composition can regulate the quantity and quality of root exudates and litter inputs, thereby altering the availability of soil carbon substrates and producing cascading effects on fungal communities (Klumpp et al., 2009; Song et al., 2022; Liu et al., 2023). Therefore, clarifying the mechanisms underlying grazing effects on fungal communities is critical for understanding how grassland ecosystems respond to anthropogenic disturbance.

Previous studies have demonstrated that grazing intensity can significantly affect fungal diversity, yet the specific response patterns remain inconsistent. For example, some studies have found that grazing significantly reduces soil fungal α-diversity, with this negative effect being more pronounced in deeper soil layers (Tu et al., 2025). In contrast, other studies have suggested that moderate grazing can maintain or even enhance fungal diversity (Wang et al., 2022c). These discrepancies may largely reflect the combined regulation of grazing intensity, grassland type, and environmental context. Meanwhile, fungal communities in different soil layers often exhibit clear vertical differentiation in their responses to grazing, and the dominant driving factors may vary among soil layers (Wu et al., 2022; Zhou et al., 2024; Tu et al., 2025). However, most existing studies have focused on a single soil layer, and systematic comparisons among different soil layers remain limited. Soil total nitrogen, nitrate nitrogen, and soil moisture have been identified as key factors regulating fungal diversity, while changes in resource availability and niche compression may further alter community structure by influencing interspecific competition (Wang et al., 2022c; Song et al., 2023; Yang et al., 2024). Overall, changes in fungal diversity are shaped by the interplay of multiple biotic and abiotic drivers. However, the relative importance of these drivers and the pathways through which they directly and indirectly regulate fungal communities remain poorly understood.

In recent years, high-throughput sequencing-based microbial co-occurrence network analysis has become an important approach for elucidating interactions within microbial communities (Faust and Raes, 2012; Zhang et al., 2018). This approach can reveal potential association patterns among taxa, and network topological properties are commonly used as indicators of microbial community organization and potential resilience under environmental disturbances (Chen et al., 2021a; Ding et al., 2023). Grazing disturbance can substantially alter the structure of soil microbial networks. For example, grazing exclusion has been reported to reduce the stability of fungal networks (Chen et al., 2020), whereas intensive animal trampling may increase bacterial network stability but weaken fungal network stability (Wang et al., 2022a). Mechanistically, grazing can affect the strength and pattern of associations among taxa by modifying resource input patterns and environmental heterogeneity. However, the mechanisms underlying the responses of fungal co-occurrence network structure and organization to grazing remain insufficiently understood. Therefore, clarifying how different grazing intensities affect the complexity, connectivity, and potential association patterns of fungal co-occurrence networks is essential for assessing the stability and sustainable management of grassland ecosystems.

However, current understanding of how grazing affects soil fungal communities has largely focused on single soil layers or changes in community diversity, while the driving mechanisms underlying fungal diversity and co-occurrence networks across different soil layers remain insufficiently explored. Based on previous studies, we selected a typical grassland in the agro-pastoral ecotone of Youyu County, Shanxi Province, China, as the study area. Specifically, this study aimed to: (1) investigate the responses of fungal α-diversity and community composition to different grazing intensities across soil layers, (2) characterize changes in fungal co-occurrence network organization under different grazing regimes, and (3) identify the potential pathways linking grazing intensity, vegetation properties, soil environmental conditions, microbial biomass, and fungal diversity using piecewise structural equation modeling. We hypothesized that (1) grazing intensity would significantly alter fungal diversity and community composition, with responses differing among soil layers due to variation in resource availability and environmental conditions; (2) fungal co-occurrence network organization would be modified by grazing intensity, reflecting shifts in potential associations among fungal taxa under grazing-induced changes in resource availability and environmental conditions, and (3) grazing intensity would be associated with variation in fungal diversity through interconnected pathways involving vegetation inputs, soil properties, and microbial biomass. This study provides insights into the sustainable management of grazing practices and the maintenance of soil fungal communities in grasslands of the agro-pastoral ecotone.

## 2 Materials and methods

### 2.1 Study area and experimental design

The experimental site is located in a typical semi-arid grassland of the agro-pastoral ecotone at the National Field Scientific Observation and Research Station of Grassland Ecosystems on the Loess Plateau (39 °59′N, 112 °19′E). The region has a mean annual temperature of 4.7°C, with precipitation mainly concentrated from May to July, and a mean annual precipitation of 435 mm. The soil texture is sandy, and the soil type is chestnut soil, with a pH ranging from 9 to 10. The dominant plant species include *Leymus secalinus, Setaria viridis, Puccinellia distans, Artemisia argyi*, and *Suaeda glauca*.

The long-term grazing experiment was established in 2016. For the present study, four grazing treatments were randomly assigned to 12 independently fenced plots (approximately 0.2 ha each), with three replicate plots per treatment. Four grazing intensity treatments were included: an ungrazed control (CK, 0 sheep units ha^−1^ growing season^−1^), light grazing (LG, 2.35 sheep units ha^−1^ growing season^−1^), moderate grazing (MG, 4.80 sheep units ha^−1^ growing season^−1^), and heavy grazing (HG, 7.85 sheep units ha^−1^ growing season^−1^). The grazing intensity levels followed the established long-term experimental design for this site. Healthy Dorper-crossbred sheep with an initial body weight of approximately 25 kg were used for grazing. One, two, and four sheep were assigned to LG, MG, and HG plots, respectively, whereas CK plots remained ungrazed. Grazing was conducted continuously from early June to late October each year, lasting approximately five months. The sheep remained in their assigned plots throughout each grazing season and were not rotated or exchanged among replicate plots.

### 2.2 Soil sample collection

Soil sampling was conducted in late June 2024 while grazing was in progress. Within each grazing plot, three sampling points were randomly selected to represent the overall vegetation conditions of the plot. After removing aboveground vegetation and undecomposed surface litter, the exposed mineral soil surface was defined as 0 cm. Soil samples were then collected from three predefined depth intervals: the surface layer (0-2 cm), middle layer (2-10 cm), and lower layer (10-20 cm), using a soil auger with a diameter of 5 cm. These terms were used to distinguish sampling depths and do not represent separate pedogenic horizons. Within each plot, the three samples collected from the same soil layer were pooled and thoroughly mixed to form one composite sample, resulting in 36 composite samples in total. These composite samples were stored at 4°C, transported to the laboratory, and subsequently used for soil chemical analyses and ITS sequencing.

### 2.3 Soil physicochemical analyses

Soil bulk density was measured by the core-ring method using undisturbed soil cores collected with a 100 cm^3^ cutting ring. The samples were oven-dried at 105°C to constant weight, and bulk density was calculated as oven-dry soil mass per unit volume (g cm^−3^). Soil pH was measured using a pH meter at a soil-to-water ratio of 1:2.5. Total carbon (TC) and total nitrogen (TN) were determined using an elemental analyzer. Soil organic carbon (SOC) and dissolved organic carbon (DOC) were measured using a total organic carbon analyzer. Available phosphorus (AP) was extracted with 0.05 mol L^−1^ NaHCO_3_ and determined using a UV spectrophotometer. The soil C:N ratio was calculated as the ratio of SOC to TN, because SOC:TN more directly reflects soil organic matter quality, microbial activity, and nutrient availability, whereas TC:TN may be biased by the presence of inorganic carbon. Nitrate nitrogen (${\text{NO}}_{3}^{-}$-N) and ammonium nitrogen (${\text{NH}}_{4}^{+}$-N) were determined using a CleverChem automated discrete chemistry analyzer. Microbial biomass carbon (MBC) and microbial biomass nitrogen (MBN) were determined using the chloroform fumigation-K_2_SO_4_ extraction method.

### 2.4 DNA extraction and high-throughput sequencing

Soil fungal community composition and diversity were analyzed using high-throughput sequencing. Total genomic DNA was extracted from soil samples using the E.Z.N.A.^®^ Soil DNA Kit (Omega Bio-tek, Norcross, GA, USA). The integrity of the extracted DNA was assessed by 1% agarose gel electrophoresis, and its concentration and purity were determined using a NanoDrop 2000 spectrophotometer (Thermo Scientific, USA). The fungal ITS region was amplified using the primers ITS1F (5′-CTTGGTCATTTAGAGGAAGTAA-3′) and ITS2R (5′-GCTGCGTTCTTCATCGATGC-3′), with the extracted DNA as the template. The PCR products were purified and used for sequencing library construction, followed by paired-end sequencing on the Illumina MiSeq platform by Majorbio Biotechnology Co., Ltd. (Shanghai, China). Sequences were quality-filtered, clustered into operational taxonomic units (OTUs) at 97% sequence similarity, and taxonomically classified using the UNITE database. Rarefaction was performed at 20,000 sequences per sample to standardize sequencing depth.

### 2.5 Statistical analysis

All data were organized using Excel 2019. Statistical analyses were primarily conducted using R software (version 4.5.1), with PCoA ordination and visualization performed using the Majorbio Cloud Platform as described below. Linear mixed-effects models were fitted using the *lmer* function in the *lmerTest* package, which extends the *lme4* framework, to evaluate the effects of grazing intensity and soil depth on soil properties, microbial biomass, and fungal α-diversity. Grazing intensity was treated as a fixed between-plot factor, soil depth was treated as a within-plot factor, and plot identity was included as a random effect to account for the non-independence of observations collected from the same plot. Estimated marginal means were calculated using the *emmeans* function in the *emmeans* package. Pairwise comparisons among grazing intensities within each soil depth and among soil depths within each grazing intensity were performed using the pairs function with Tukey adjustment for multiple comparisons. Differences were considered significant at *P* < 0.05.

Fungal α-diversity was evaluated using the ACE, Chao1, and Shannon indices. Principal coordinate analysis (PCoA) based on Bray-Curtis dissimilarity was performed at the OTU level using the Majorbio Cloud Platform to visualize variation in fungal community composition. PERMANOVA was subsequently performed in R using the *adonis2* function in the *vegan* package to evaluate the effects of grazing intensity, soil depth, and their interaction on fungal community composition. Restricted permutation designs were generated using the *how* function in the *permute* package, with *Plots* and *Within* used to specify the whole-plot permutation structure where appropriate. Because grazing intensity was a between-plot factor whereas soil depth was a within-plot factor, separate restricted permutation schemes were used for the corresponding effects. For the grazing-intensity main effect, whole plots were permuted as intact units while preserving the three soil-depth observations within each plot. For soil depth and the grazing intensity × soil depth interaction, permutations were restricted within plot identity so that observations were permuted only among soil depths within the same plot. A total of 9,999 permutations were used for each test.

Fungal co-occurrence networks were constructed separately for each grazing intensity using OTU abundance data from the three soil depths and three replicate plots (n = 9 samples per grazing treatment). OTUs with relative abundances ≥ 0.05% and occurring in at least 60% of the samples within each grazing treatment were retained for network construction. Pairwise Spearman correlation coefficients were calculated among the retained OTUs. Network edges were retained when |r| ≥ 0.45 and *P* < 0.05. The resulting networks were constructed and visualized in R using the *igraph* and *ggraph* packages. Network topological properties, including the number of nodes and edges, average degree, modularity, average clustering coefficient, and average path length, were calculated using the *igraph* package. The proportion of positive associations was calculated as the percentage of retained edges with positive Spearman correlation coefficients. Because each grazing treatment contained only three independent plots, network topological properties were compared descriptively among grazing treatments.

Composite variables were generated using principal component analysis (PCA) implemented with the *prcomp* function in the *stats* package. Before PCA, all variables were standardized to zero mean and unit variance. The first principal component (PC1) was retained as the integrated index for each variable group. Soil C was constructed from SOC and DOC, AGBLB from aboveground biomass and litter biomass, MBCN from microbial biomass carbon and nitrogen, and fungal diversity (FD) from ACE, Chao1, and Shannon indices. Soil N was calculated as the sum of ${\text{NH}}_{4}^{+}$-N and ${\text{NO}}_{3}^{-}$-N and represented total inorganic nitrogen. The indicators included in each PCA-derived composite variable and the corresponding PC1 explanatory percentages are provided in Supplementary Table 2.

Piecewise structural equation modeling (piecewise SEM) was conducted using the *psem* function in the *piecewiseSEM* package to evaluate direct and indirect associations among grazing intensity, vegetation biomass, soil properties, microbial biomass, and fungal diversity. The component models consisted of linear mixed-effects models fitted using the *lme4* package and ordinary linear models fitted using the *stats* package. Grazing intensity was entered as a numeric treatment score representing increasing grazing pressure (CK = 0, LG = 1, MG = 2, and HG = 4). Soil depth was included as a categorical fixed effect, and plot identity was included as a random effect where appropriate. An initial candidate model included belowground biomass (BGB) and soil C:N; these terms were removed from the final reduced model because they did not contribute significantly to the relevant component models, while the overall model interpretation remained unchanged. A residual correlation between Soil N and soil pH was included in the piecewise SEM to account for their remaining association after considering the specified model pathways. Standardized path coefficients were obtained using *coefs*, coefficients of determination (R^2^) were obtained using *rsquared*, and overall model fit was evaluated using *fisherC*, with *P* > 0.05 indicating adequate model fit. Because aboveground biomass and litter biomass were measured at the plot level, the GI-AGBLB relationship was fitted separately using one observation per independent plot (n = 12), whereas the soil-depth component models used all 36 observations.

## 3 Results

Statistical analyses showed that grazing intensity, soil depth, and their interaction affected soil physicochemical properties to different degrees (Figure 1; Supplementary Table 3). Grazing intensity significantly affected DOC, whereas soil depth significantly affected SOC, TC, and TN; neither the other main effect nor their interaction was significant for these variables. DOC differences among grazing treatments were mainly observed in the upper two soil layers, with lower values under HG than under MG in the 0–2 cm layer and under CK in the 2–10 cm layer. SOC, TC, and TN generally showed clear vertical stratification, with higher contents in the 0–2 cm layer than in the deeper layers. ${\text{NH}}_{4}^{+}$-N and ${\text{NO}}_{3}^{-}$-N were significantly affected by grazing intensity, soil depth, and their interaction. In the 0-2 cm layer, ${\text{NH}}_{4}^{+}$-N was significantly higher under MG than under the other grazing treatments, whereas ${\text{NO}}_{3}^{-}$-N was generally higher under LG than under the other treatments across soil layers. The C:N ratio was also significantly affected by grazing intensity, soil depth, and their interaction, with grazing-related differences occurring mainly in the 0–2 and 2–10 cm layers and no significant differences among grazing treatments in the 10–20 cm layer. AP was significantly affected by soil depth and the grazing intensity × soil depth interaction, but not by grazing intensity alone; in the 0–2 cm layer, AP was significantly higher under MG and HG than under LG. Detailed pairwise comparisons are indicated by the letter codes in Figure 1.

**Figure 1:**
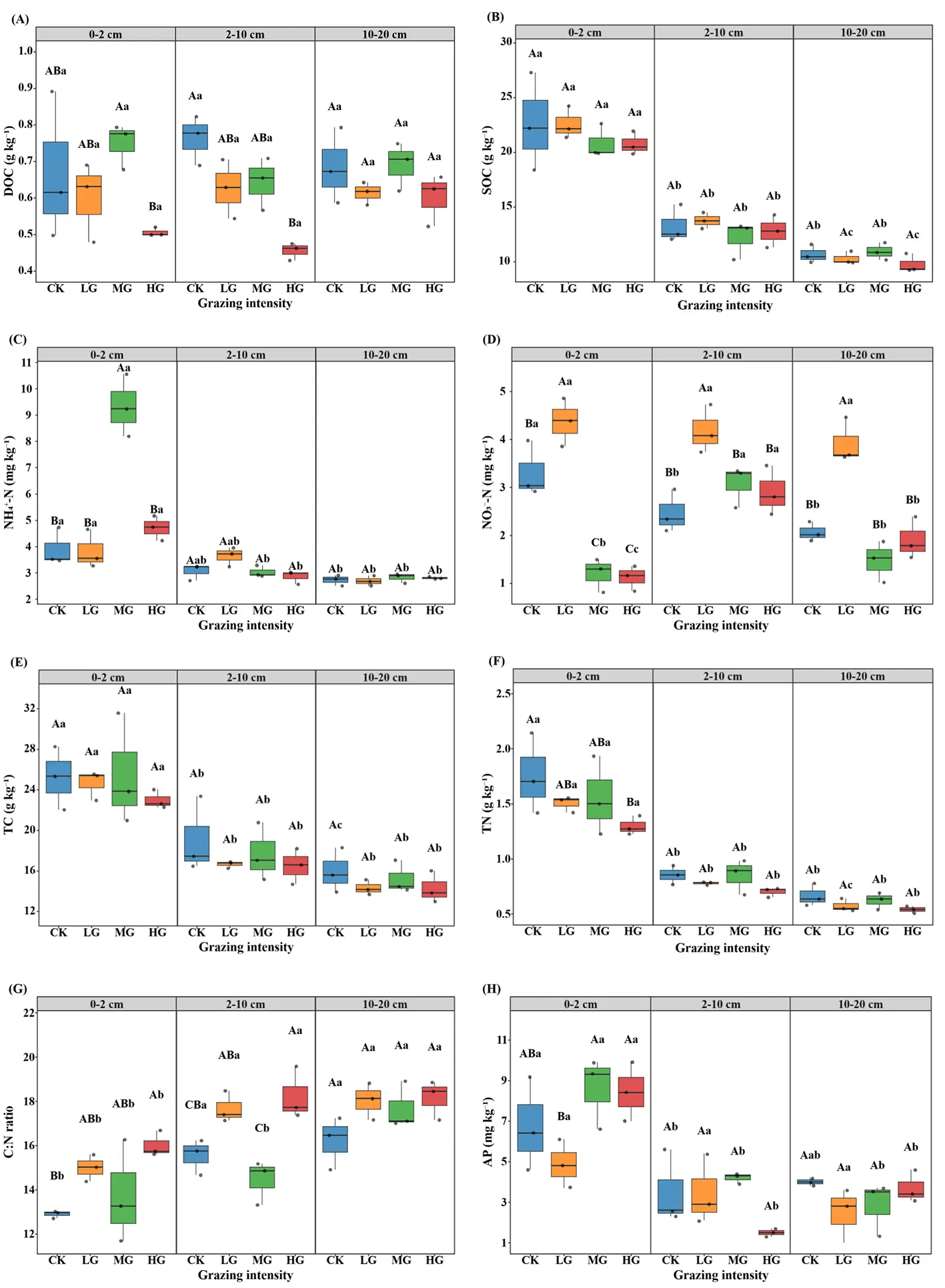
Effects of grazing intensity and soil depth on soil physicochemical properties. Panels A-H represent: **(A)** DOC, dissolved organic carbon; **(B)** SOC, soil organic carbon; **(C)** ${\text{NH}}_{4}^{+}$-N, ammonium nitrogen; **(D)** ${\text{NO}}_{3}^{-}$-N, nitrate nitrogen; **(E)** TC, total carbon; **(F)** TN, total nitrogen; **(G)** C:N, soil carbon-to-nitrogen ratio; and **(H)** AP, available phosphorus. Different uppercase letters indicate significant differences among grazing intensities within the same soil layer, whereas different lowercase letters indicate significant differences among soil layers under the same grazing intensity (*P* < 0.05). Statistical comparisons were performed using linear mixed models followed by estimated marginal means comparisons. Gray points represent individual observations from each plot (*n* = 3 per grazing intensity × soil layer combination).

Soil pH was significantly affected by grazing intensity, soil depth, and their interaction (Figure 2; Supplementary Table 3). Differences among grazing treatments were mainly observed in the 0–2 and 2–10 cm soil layers. In the 0–2 cm layer, soil pH was significantly higher under HG than under CK, LG, and MG, whereas in the 2–10 cm layer, HG also showed significantly higher pH than the other grazing treatments. In contrast, no significant differences among grazing treatments were observed in the 10–20 cm layer. Soil pH also varied significantly with depth, although the vertical pattern differed among grazing treatments. Detailed pairwise comparisons are indicated by the letter codes in Figure 2. Soil bulk density was significantly affected by soil depth, whereas grazing intensity and the grazing intensity × soil depth interaction had no significant effects (Supplementary Figure 1; Supplementary Table 3).

**Figure 2:**
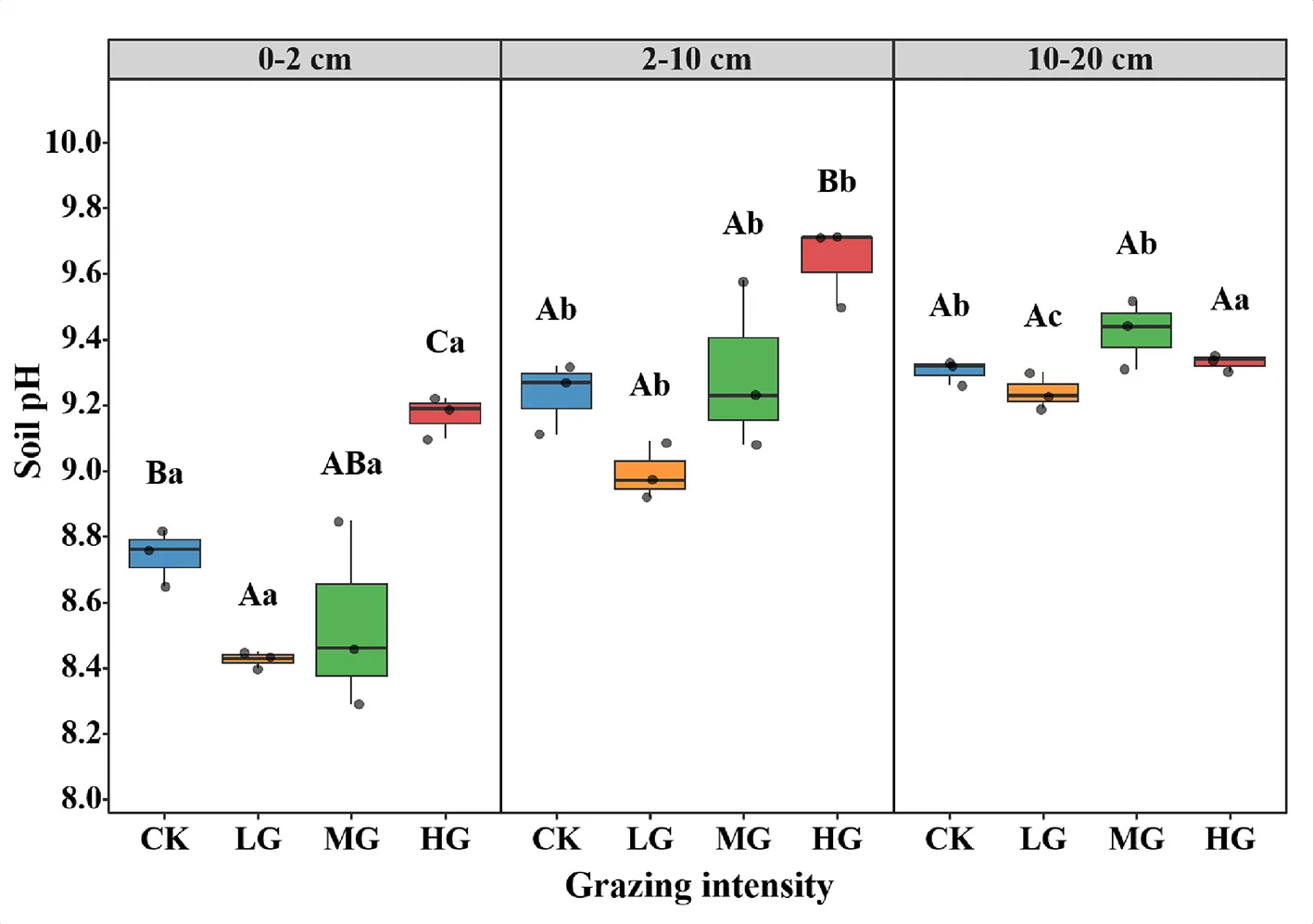
Effects of grazing intensity and soil depth on soil pH. CK, ungrazed control; LG, light grazing; MG, moderate grazing; HG, heavy grazing. Soil layers were classified as surface soil (0–2 cm), middle soil (2-10 cm), and lower soil (10-20 cm). Different uppercase letters indicate significant differences among grazing intensities within the same soil layer, whereas different lowercase letters indicate significant differences among soil layers within the same grazing intensity (*P* < 0.05). Statistical comparisons were performed using linear mixed models followed by estimated marginal means comparisons. Gray points represent individual observations from each plot (*n* = 3 per grazing intensity × soil layer combination).

Soil depth significantly affected both microbial biomass carbon (MBC) and microbial biomass nitrogen (MBN), whereas neither grazing intensity nor the grazing intensity × soil depth interaction had significant effects (Figure 3; Supplementary Table 3). Within the same soil layer, neither MBC nor MBN differed significantly among grazing treatments. MBC was consistently higher in the 0–2 cm layer than in the 2–10 and 10–20 cm layers across all grazing treatments. MBN showed a similar vertical pattern, with the highest values generally occurring in the 0–2 cm layer. Detailed pairwise comparisons among soil layers are indicated by the letter codes in Figure 3.

**Figure 3:**
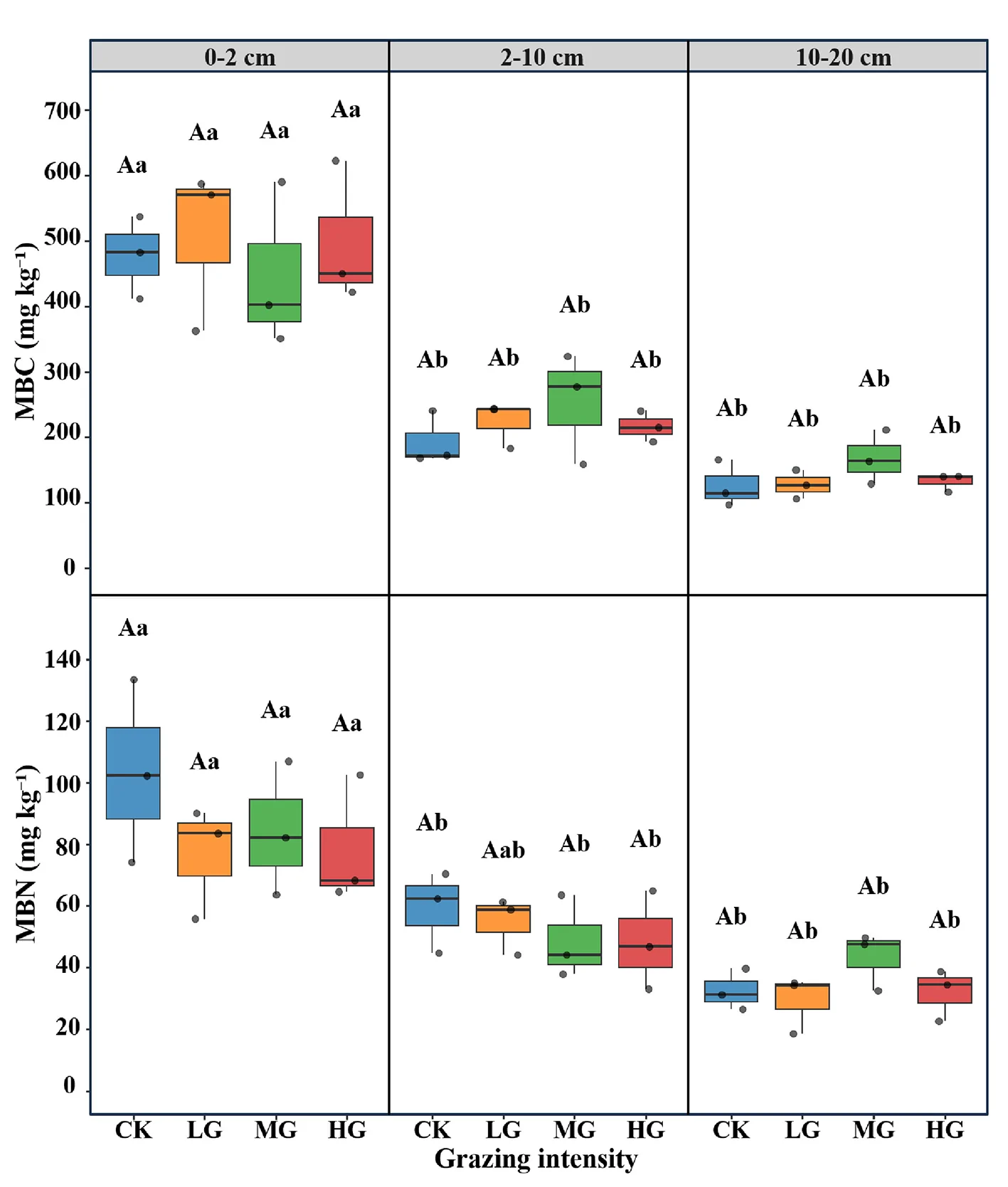
Effects of grazing intensity and soil depth on soil microbial biomass carbon (MBC) and microbial biomass nitrogen (MBN). Soil layers were classified as surface soil (0–2 cm), middle soil (2–10 cm), and lower soil (10–20 cm). Different uppercase letters indicate significant differences among grazing intensities within the same soil layer, whereas different lowercase letters indicate significant differences among soil layers within the same grazing intensity (*P* < 0.05). Statistical comparisons were performed using linear mixed models followed by estimated marginal means comparisons. Gray points represent individual observations from each plot (*n* = 3 per grazing intensity × soil layer combination).

Soil fungal α-diversity showed index-specific and depth-dependent responses to grazing intensity (Figures 4A–C; Supplementary Table 3). Soil depth and the grazing intensity × soil depth interaction significantly affected both ACE and Chao1, whereas the main effect of grazing intensity was not significant. In the 0-2 cm layer, ACE and Chao1 were significantly higher under LG and MG than under HG, while no significant differences among grazing treatments were observed in the 2–10 or 10–20 cm layers. Across grazing treatments, both richness indices were generally higher in the 0–2 cm layer than in the deeper soil layers. In contrast, Shannon diversity was not significantly affected by grazing intensity, soil depth, or their interaction, indicating a weaker response than ACE and Chao1. Detailed pairwise comparisons are indicated by the letter codes in Figures 4A–C.

**Figure 4:**
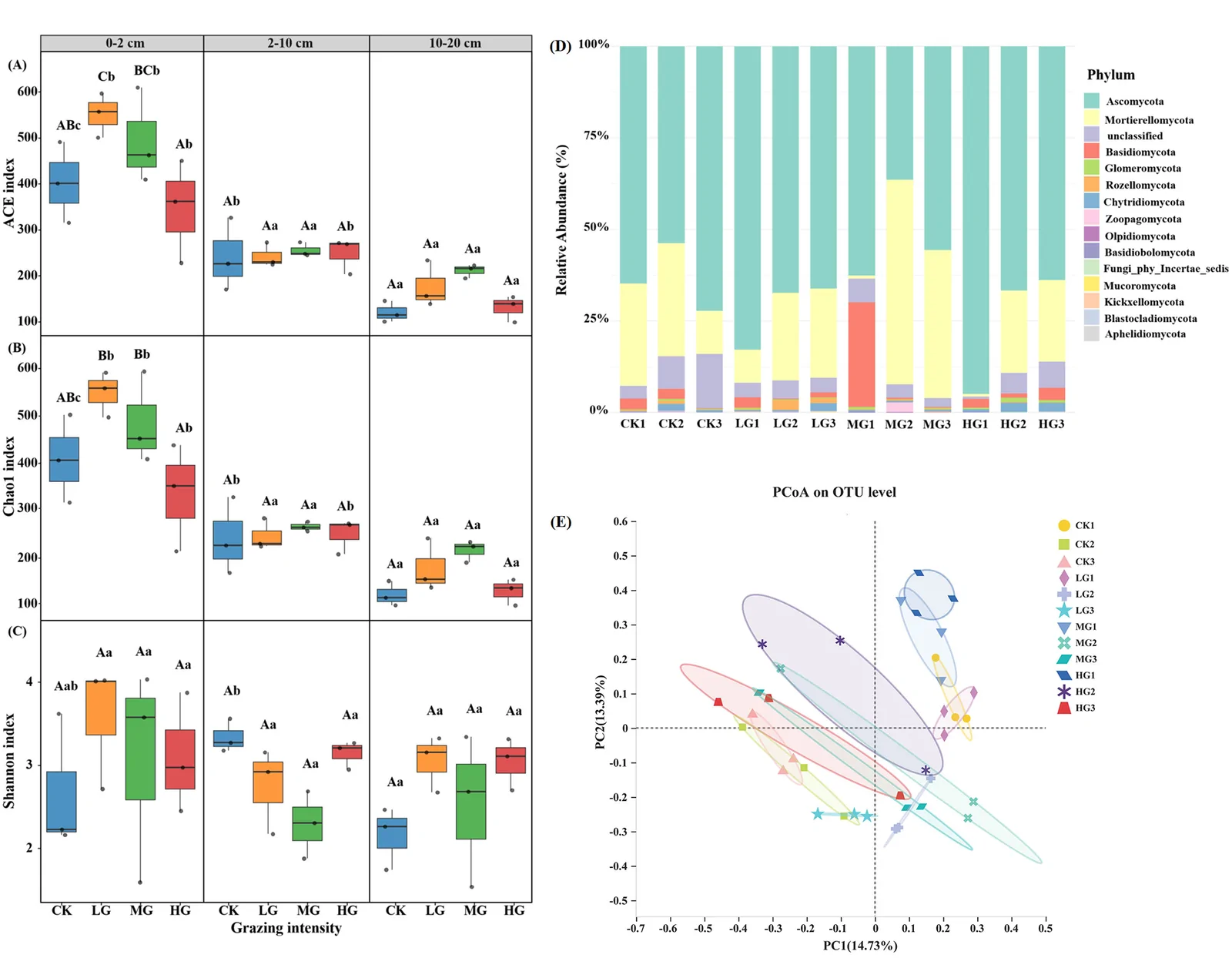
Responses of soil fungal diversity and community composition to grazing intensity across soil layers. **(A)** ACE, **(B)** Chao1, and **(C)** Shannon diversity indices under different grazing intensities across the surface (0–2 cm), middle (2–10 cm), and lower (10–20 cm) soil layers. **(D)** Relative abundances of the 15 most abundant fungal phyla across grazing treatments and soil layers. **(E)** Principal coordinate analysis (PCoA) of fungal community composition based on Bray-Curtis dissimilarity at the OTU level. The numerals 1, 2, and 3 following the treatment abbreviations in panels D and E represent the surface (0–2 cm), middle (2–10 cm), and lower (10–20 cm) soil layers, respectively. In panels A-C, different uppercase letters indicate significant differences among grazing intensities within the same soil layer, whereas different lowercase letters indicate significant differences among soil layers within the same grazing intensity (*P* < 0.05). Statistical comparisons in panels A-C were performed using linear mixed-effects models followed by estimated marginal means comparisons. Gray points represent individual plot-level observations (n = 3 per grazing intensity × soil layer combination). Each stacked bar in panel D represents the mean relative abundance of three independent plots. Factorial PERMANOVA in panel E evaluated the effects of grazing intensity, soil depth, and their interaction on community dissimilarity using 9,999 permutations. Whole-plot permutations were used to test the grazing-intensity main effect, whereas permutations for soil depth and the grazing intensity × soil depth interaction were restricted within plots to account for repeated observations across soil depths. Ellipses represent 95% confidence intervals. Panel E includes all 36 composite soil samples collected from 12 independent plots across three soil depths.

At the phylum level, the relative abundances of the 15 most abundant fungal phyla are presented (Figure 4D). Under CK, Ascomycota and Mortierellomycota accounted for relatively large proportions across all three soil layers, with relative abundances of 64.78% and 27.99% in the 0–2 cm layer, 53.77% and 30.86% in the 2–10 cm layer, and 72.22% and 11.76% in the 10–20 cm layer, respectively. Similarly, under LG, the relative abundances of Ascomycota and Mortierellomycota were 82.85% and 9.09%, 67.29% and 24.03%, and 66.19% and 24.31% in the 0–2 cm, 2–10 cm, and 10–20 cm layers, respectively. Under MG, Ascomycota and Basidiomycota accounted for 62.62% and 28.53%, respectively, in the 0–2 cm layer. In the 2–10 cm layer, Mortierellomycota accounted for the largest proportion (55.90%), followed by Ascomycota (36.49%), whereas their relative abundances were 40.38% and 55.70%, respectively, in the 10–20 cm layer. Under HG, Ascomycota accounted for 94.94%, 66.74%, and 63.84% in the 0–2 cm, 2–10 cm, and 10–20 cm layers, respectively. Mortierellomycota accounted for 22.44% in the 2–10 cm layer and 22.29% in the 10–20 cm layer, whereas Basidiomycota accounted for 3.32% in the 10–20 cm layer. The relative abundances of fungal classes, orders, families, and genera are presented in Supplementary Figures 2–5, respectively.

PCoA ordination at the OTU level showed variation in soil fungal community composition among grazing treatments and soil depths (Figure 4E). PC1 and PC2 explained 14.73% and 13.39% of the total community variation, respectively, together accounting for 28.12%. Factorial PERMANOVA based on Bray-Curtis dissimilarity showed significant effects of grazing intensity (*R*^2^ = 0.145, *P* = 0.043) and soil depth (*R*^2^ = 0.151, *P* < 0.001) on fungal community composition, whereas the grazing intensity × soil depth interaction was not significant (*R*^2^ = 0.125, *P* = 0.411). In the ordination space, CK and LG samples were more frequently distributed toward the negative side of PC1, whereas MG and HG samples occurred more frequently toward the positive side, although substantial overlap was present among grazing treatments. Samples from different soil depths also showed partial separation, with considerable overlap among depth groups.

To examine the changes in fungal co-occurrence patterns under different grazing intensities, co-occurrence networks were constructed for each grazing treatment, and their topological properties were compared (Figure 5; Supplementary Table 1). The network structures showed variation among grazing intensities in network size, connectivity, and topological organization.

**Figure 5:**
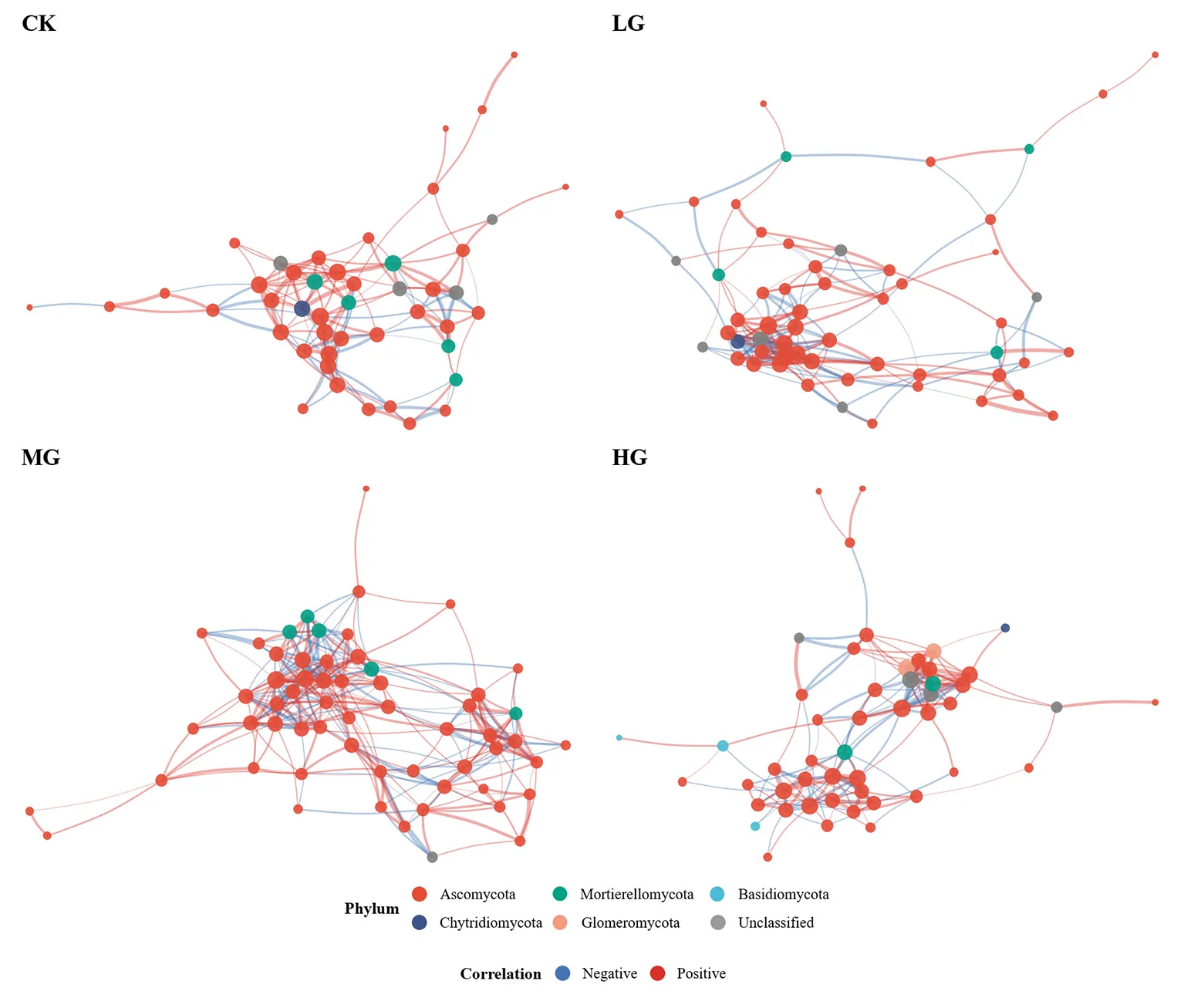
Co-occurrence networks of soil fungal communities under different grazing intensities. CK, ungrazed control; LG, light grazing; MG, moderate grazing; HG, heavy grazing. Each network was constructed based on pairwise Spearman correlations among fungal OTUs within each grazing intensity (*n* = 9 samples), with edges retained at |r| ≥ 0.45 and *P* < 0.05. Node size represents degree, node color indicates fungal phylum classification, and red and blue edges indicate positive and negative associations, respectively. Edge width represents the absolute strength of the Spearman correlation.

The network size varied substantially among grazing treatments. The MG network contained the highest number of nodes and edges (59 nodes and 297 edges), followed by LG (56 nodes and 205 edges), HG (50 nodes and 187 edges), and CK (46 nodes and 167 edges). Consistently, the average degree was highest under MG (10.068), whereas CK, LG, and HG showed relatively lower and similar values (7.261, 7.321, and 7.480, respectively). Network modularity and clustering properties also varied among grazing treatments. Modularity values ranged from 0.394 to 0.440, with the highest value observed under HG (0.440) and the lowest under MG (0.394). The average clustering coefficient ranged from 0.492 to 0.571, with HG showing the highest value (0.571), followed by MG (0.551), CK (0.514), and LG (0.492). Average path length varied from 2.330 to 2.986, with LG exhibiting the longest path length and MG exhibiting the shortest. Positive correlations accounted for 50.73–65.87% of total correlations across grazing treatments. CK showed the highest proportion of positive correlations (65.87%), whereas LG showed the lowest proportion (50.73%). Negative correlations accounted for 34.13-49.27% of total correlations and varied among grazing treatments.

Piecewise structural equation modeling (piecewise SEM) was conducted to evaluate the associations among grazing intensity, plant biomass, soil properties, microbial biomass, and fungal diversity (Figure 6). The final model showed a good fit to the data (Fisher's C = 21.77, df = 30, *P* = 0.862). The model explained 54%, 59%, 58%, 68%, 88%, and 68% of the variance in Soil C, Soil N, AP, soil pH, MBCN, and fungal diversity, respectively. Grazing intensity showed significant associations with plant biomass and soil properties. Grazing intensity was negatively associated with AGBLB and Soil N, whereas it showed a positive association with soil pH. In addition, AGBLB was negatively associated with Soil N. Grazing intensity and AGBLB together explained 59% of the variation in Soil N. Soil properties were important predictors of microbial biomass. Soil C and soil pH showed positive associations with MBCN, and these variables jointly explained 88% of the variation in MBCN. Fungal diversity showed significant associations with Soil C, AP, and MBCN. Soil C was positively associated with fungal diversity, whereas AP and MBCN showed negative associations with fungal diversity. These variables jointly explained 68% of the variation in fungal diversity.

**Figure 6:**
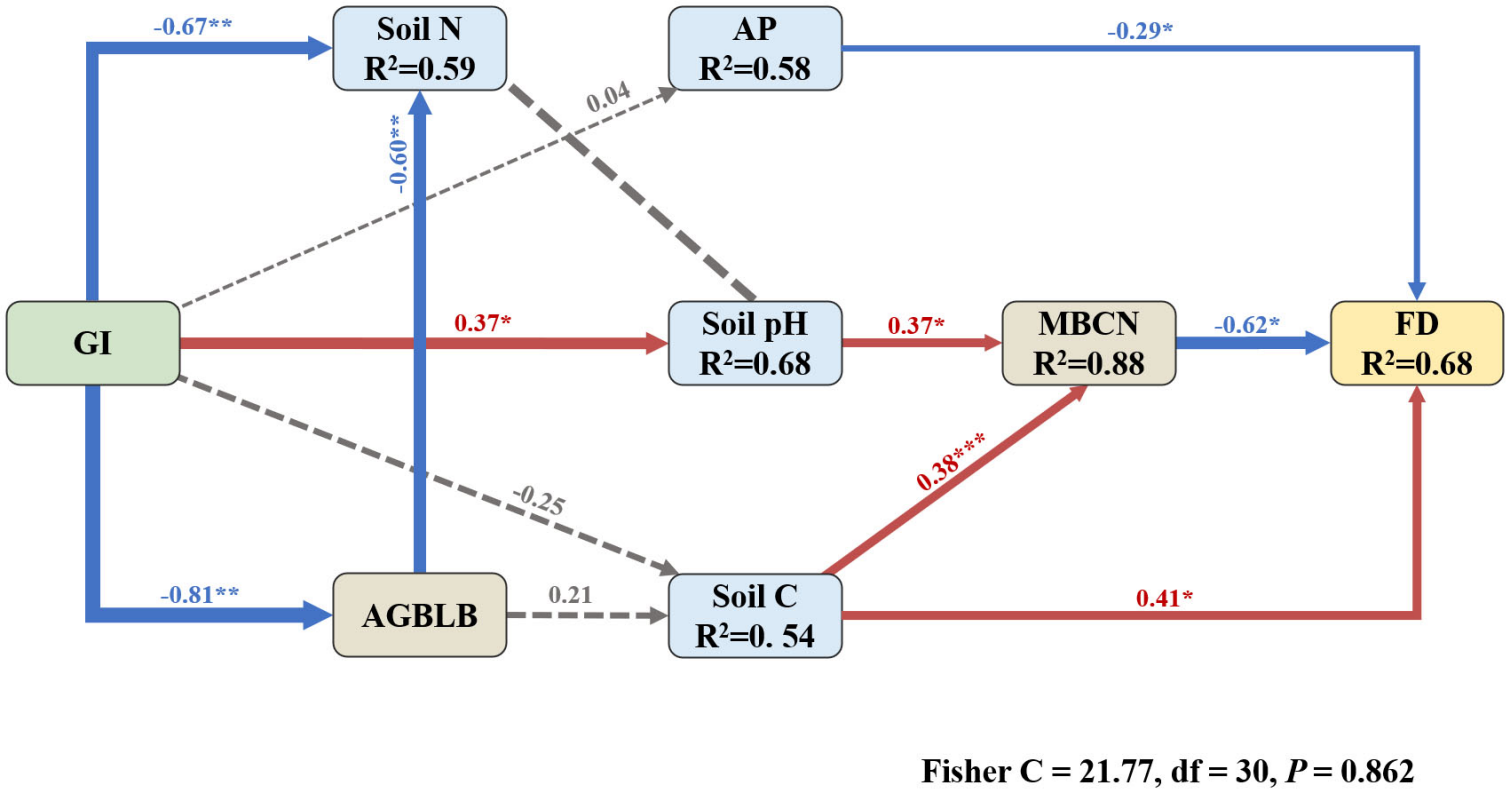
Piecewise structural equation model illustrating associations among grazing intensity, vegetation biomass, soil properties, microbial biomass, and soil fungal diversity. Numbers adjacent to directional arrows represent standardized path coefficients. Red and blue solid arrows indicate significant positive and negative associations, respectively, whereas gray dashed arrows indicate non-significant pathways. The dashed line without arrowheads between Soil N and soil pH represents their correlation (*r* = −0.41) rather than a directional pathway. Asterisks indicate the significance levels of standardized path coefficients: ***P < 0.001, **P < 0.01, and *P < 0.05. Values within the boxes of endogenous variables represent coefficients of determination (R^2^). Overall model fit was evaluated using Fisher's C test (Fisher's *C* = 21.77, df = 30, *P* = 0.862). Soil C, AGBLB, MBCN, and fungal diversity (FD) are composite variables constructed using principal component analysis (PCA). Soil C was constructed from dissolved organic carbon (DOC) and soil organic carbon (SOC); AGBLB from aboveground biomass and litter biomass; MBCN from microbial biomass carbon (MBC) and microbial biomass nitrogen (MBN); and FD from the ACE, Chao1, and Shannon indices. Soil N represents total inorganic nitrogen and was calculated as the sum of ${\text{NH}}_{4}^{+}$-N and ${\text{NO}}_{3}^{-}$-N. The soil-depth component models included 36 observations from 12 independent plots across three soil depths, with plot identity included as a random effect where appropriate; the GI-AGBLB relationship was fitted separately at the plot level using one observation per plot (*n* = 12).

## 4 Discussion

### 4.1 Soil layer-dependent responses of fungal community composition and diversity along the grazing intensity gradient

We examined patterns in the relative abundance of fungal phyla across grazing intensities. The results showed that Ascomycota and Mortierellomycota accounted for relatively large proportions of the fungal community across most treatment-by-depth combinations, whereas Basidiomycota showed a relatively large proportion only in some combinations (Figure 4D). Previous studies have also reported that these phyla are commonly abundant in different grassland ecosystems, suggesting that they may have strong environmental adaptability and competitive advantages (Zhao et al., 2023; Xiang et al., 2025). Among these phyla, Ascomycota maintained high relative abundance across all grazing treatments, which may be related to its strong environmental tolerance (Zhao et al., 2023; Manici et al., 2024). Members of this phylum can produce large numbers of conidia, enhancing their ability to tolerate adverse environmental conditions such as drought, high temperature, and ultraviolet radiation (Hagiwara et al., 2017). Given the strongly alkaline conditions at our study site (pH 9-10), high soil pH may also have acted as an environmental filter, favoring Ascomycota taxa with alkaliphilic or alkali-tolerant traits and thereby contributing to their high relative abundance. In addition, many Ascomycota taxa are saprotrophic fungi that can rapidly utilize dissolved organic carbon in soil and maintain high metabolic activity under fluctuating nutrient conditions (Manici et al., 2024; Yang et al., 2024). Grazing-induced changes in soil pH, organic matter, and nutrient availability may further enhance their competitive advantage for resources, thereby helping Ascomycota maintain its relatively high abundance (Wang et al., 2022c; Qin et al., 2023; Xu et al., 2023). Mortierellomycota is closely associated with soil carbon cycling and often responds to changes in soil nutrient conditions (Ozimek and Hanaka, 2021; Hu et al., 2024). Its relatively high abundance in the 2-10 cm layer under MG may therefore reflect the combined influence of resource availability and other soil environmental conditions under this treatment.

The relative abundance patterns of the major fungal phyla varied among treatment and depth combinations. Under MG, Ascomycota and Basidiomycota accounted for relatively large proportions in the 0–2 cm layer, whereas Mortierellomycota accounted for the largest proportion in the 2–10 cm layer (Figure 4D). This pattern may be associated with differences in grazing disturbance and resource distribution among soil layers (Zhou et al., 2024; Tu et al., 2025). The surface soil is directly affected by livestock trampling and fecal inputs, which can more readily alter soil aeration, nutrient distribution, and organic matter input (Wang et al., 2022c; Yang et al., 2022). These changes may lead to differential responses among fungal taxa in resource utilization and environmental adaptation. Basidiomycota are often involved in the decomposition of complex organic compounds such as lignin and are relatively sensitive to changes in soil pH and nutrient availability (Averill and Waring, 2018; Manici et al., 2024). In the 0–2 cm soil layer under MG, the relatively high abundance of Basidiomycota may be associated with soil pH, nutrient availability, and organic matter conditions. In addition, grazing may further affect fungal community structure by altering soil nitrogen content and the C:N ratio. Higher nitrogen input often favors some fast-growing fungal taxa, whereas it may suppress taxa that depend on more complex substrates, thereby limiting the development of Basidiomycota (Averill and Waring, 2018; Feng et al., 2024; Yang et al., 2024). These factors may partly explain the observed differences in the relative abundances of fungal phyla among soil layers under MG.

At the family level, different fungal taxa showed more pronounced functional differentiation, which was closely associated with soil nutrient conditions. Mortierellaceae and its corresponding genus 属名斜体 maintained relatively high proportions across several treatments (Supplementary Figure 4). These fungi are typical saprotrophs that can decompose complex organic compounds such as cellulose and chitin and promote the release of nutrients such as phosphorus and iron in soil, thereby increasing nutrient availability (Ozimek and Hanaka, 2021; Hu et al., 2024). Nectriaceae and its representative genus 属名斜体 include both saprotrophic taxa and plant pathogens. These fungi often increase under conditions of nutrient fluctuation or environmental disturbance, and their changes in abundance may reflect shifts in soil environmental conditions and resource status (Hu et al., 2024). In addition, some taxa within Cladosporiaceae and Phaeosphaeriaceae include plant pathogen-related species, and their relative abundances are closely associated with changes in soil nutrients and pH (Hu et al., 2024).

At the genus level, 属名斜体 maintained relatively high abundance across several soil layers (Supplementary Figure 5). Many members of this genus are saprotrophic fungi involved in decomposition, soil carbon cycling, and nutrient transformation (Ozimek and Hanaka, 2021). 属名斜体 are typical entomopathogenic fungi, and variation in their abundance may be related to shifts in soil biological interactions under grazing (Lovett and St. Leger, 2017). Relatively high abundances of 属名斜体 were observed under high grazing intensity. These genera include saprotrophic and plant pathogenic taxa and can maintain growth under nutrient fluctuations and disturbance conditions (Hu et al., 2024). Their relatively high abundances under HG may therefore reflect changes in fungal groups associated with organic matter turnover and plant health (Liao et al., 2021; Hu et al., 2024).

The effects of grazing intensity on soil fungal α-diversity were strongly dependent on soil layer. In this study, the ACE and Chao1 indices were significantly higher under LG and MG than under HG in the 0–2 cm soil layer (Figures 4A, B), suggesting that light to moderate grazing may better maintain fungal richness in surface soil than heavy grazing (Yao et al., 2023; Xiang et al., 2025). This may be because light grazing increases the availability of organic substrates through livestock fecal return, plant residue turnover, and rhizosphere inputs (Song et al., 2022; Wang et al., 2022c), thereby enhancing resource heterogeneity and providing ecological niches for a wider range of fungal taxa. In contrast, heavy grazing may reduce aboveground plant biomass and litter input while increasing soil compaction, thereby limiting the growth and colonization of some fungal taxa (Yang et al., 2022; Xu et al., 2023). Unlike the ACE and Chao1 indices, the Shannon index showed relatively small changes among grazing intensities (Figure 4C), indicating that grazing primarily affected fungal species richness, whereas its effect on community evenness was limited (Yao et al., 2023; Xiang et al., 2025). In addition, fungal diversity showed clear vertical differentiation across soil layers. Across soil layers, fungal richness showed treatment-dependent vertical patterns. Under CK, ACE and Chao1 decreased significantly with increasing soil depth, whereas under LG and MG, the surface layer showed higher richness than the deeper layers. Under HG, ACE and Chao1 were significantly lower in the 10–20 cm layer than in the upper two layers, suggesting that grazing intensity modified the vertical distribution pattern of fungal richness (Wu et al., 2022; Hu et al., 2023). Because the surface layer receives greater inputs of litter residues, root-derived carbon, and grazing-related organic inputs, its higher fungal richness may reflect greater resource availability and substrate heterogeneity relative to deeper layers (Song et al., 2022; Xu et al., 2023). By comparison, resource inputs generally decline with increasing soil depth, and the influence of plant root activity and grazing disturbance becomes weaker, which may explain the lower fungal richness in the 2-10 cm and 10-20 cm soil layers (Hu et al., 2023). Notably, the differences among soil layers were weakened under HG, suggesting that heavy grazing may reduce the resource advantage of the surface soil (Yang et al., 2022; Li et al., 2025), thereby weakening the vertical differentiation of fungal communities. Overall, grazing effects on fungal richness were dependent on grazing intensity and soil depth, with moderate grazing showing relatively higher richness in surface soil, whereas heavy grazing was associated with reduced fungal richness in the surface layer.

The PCoA results showed variation in soil fungal community structure among grazing intensities (Figure 4E), consistent with the significant overall effect of grazing intensity detected by PERMANOVA. CK and HG showed relatively greater separation in the ordination space, suggesting that fungal community composition differed between ungrazed and heavily grazed conditions. In contrast, LG was more closely clustered with CK, suggesting that fungal community structure under light grazing remained relatively close to the ungrazed state (Chen et al., 2021a; Zhang et al., 2025). The ordination pattern suggested greater compositional differences from the ungrazed condition at higher grazing intensities (Zhang et al., 2023; Ma et al., 2024). This differentiation may have been jointly driven by grazing-induced changes in soil physicochemical properties and plant-derived inputs (Xu et al., 2023). Under CK, higher vegetation cover and greater organic matter input may have maintained a relatively resource-rich soil environment, thereby supporting a more consistent fungal community structure. In contrast, under HG, reduced plant biomass, decreased litter input, and increased soil compaction may have lowered organic matter content and resource availability, potentially selecting for fungal taxa better adapted to nutrient-limited and disturbed conditions and consequently reshaping community structure. The relatively close position of LG and CK in the ordination space may reflect a comparatively limited compositional shift under light grazing. In addition, soil moisture, pH, and nutrient availability may also have jointly contributed to fungal community assembly. Given that the grazing treatments had been maintained annually since 2016, the observed community patterns may also reflect cumulative responses to repeated grazing.

### 4.2 Variation in soil fungal co-occurrence network topology across grazing intensities

Non-random co-occurrence network analysis can help reveal potential association patterns among microbial taxa under environmental change and their implications for community structure (Zhang et al., 2018). In this study, soil fungal co-occurrence network topology varied among grazing intensities, suggesting corresponding variation in potential association patterns among fungal taxa. The size and connectivity of fungal networks also varied across grazing treatments. The MG network exhibited the highest number of nodes, edges, and average degree, suggesting that moderate grazing was associated with a more complex co-occurrence pattern among fungal taxa (Figure 5). Moderate disturbance may increase spatial and resource heterogeneity by altering plant inputs, soil nutrient availability, and microenviromental conditions, thereby providing diverse ecological niches for fungal taxa and facilitating the formation of more complex co-occurrence patterns (Camuy-Vélez et al., 2024; Xiang et al., 2025). In addition, HG showed the highest modularity, whereas LG exhibited the longest average path length, suggesting a more compartmentalized network structure with reduced connectivity among fungal taxa (Faust and Raes, 2012; Zhou et al., 2025). These changes may be associated with reductions in plant biomass and litter inputs, together with increased soil compaction under heavy grazing, which could reduce resource availability and strengthen environmental filtering during fungal community assembly (Li et al., 2022). Positive associations dominated across all fungal co-occurrence networks, suggesting that fungal taxa were primarily organized through shared environmental preferences or similar responses to habitat conditions. However, the balance between positive and negative associations varied among grazing treatments, with grazing treatments showing a relatively lower proportion of positive associations than the ungrazed control, indicating shifts in potential co-occurrence patterns under grazing disturbance. Such changes may reflect altered niche differentiation and environmental filtering caused by grazing-induced changes in resource availability and soil conditions (Deutschmann et al., 2021; Xiang et al., 2025). Overall, fungal co-occurrence network topology varied across grazing intensities, with moderate grazing associated with greater network complexity and heavy grazing associated with a more modular network structure. These patterns may reflect differences in resource availability and environmental conditions among grazing regimes (Camuy-Vélez et al., 2024; Jiang et al., 2026).

### 4.3 Plant-soil-microbial pathways associated with fungal diversity under grazing

The structural equation model indicated indirect associations between grazing intensity and soil fungal diversity involving plant biomass, soil properties, and microbial biomass. Grazing intensity showed significant associations with AGBLB, Soil N, and soil pH, indicating that variation in fungal diversity was closely linked to vegetation inputs and soil environmental conditions. This pattern is consistent with previous studies showing that grazing can regulate soil microbial communities by altering plant productivity, nutrient availability, and soil physicochemical properties (Wang et al., 2022c; Kinsbergen et al., 2025). Grazing intensity was negatively associated with AGBLB and Soil N, suggesting that increased grazing pressure may reduce plant-derived inputs and alter soil nitrogen availability. Reduced aboveground biomass and litter accumulation under grazing can decrease organic matter inputs to soil, thereby affecting substrate availability for microorganisms (Xu et al., 2023). Meanwhile, grazing-induced changes in plant growth and nutrient cycling processes may modify soil nitrogen pools and alter microbial resource conditions (He et al., 2020). In contrast, grazing intensity showed a positive association with soil pH, which may be related to the redistribution of nutrients and alkaline substances through livestock excreta deposition and changes in vegetation composition (Wang et al., 2022c). These changes in vegetation and soil conditions provide an important environmental context for variation in fungal community assembly.

Soil carbon and soil pH were important predictors of microbial biomass, with both variables showing positive associations with MBCN. Soil carbon represents a major energy source for soil microorganisms, and increased carbon availability may support microbial growth by providing more accessible substrates (Wang et al., 2022b; Xu et al., 2023). In addition, soil pH acts as an important environmental constraint by modifying microbial habitat suitability and nutrient availability, thereby influencing microbial biomass accumulation (Zhang et al., 2020). The strong explanatory power of Soil C and soil pH for MBCN highlights the importance of resource availability and soil environmental conditions in explaining variation in microbial biomass under grazing disturbance. Fungal diversity was positively associated with Soil C but negatively associated with MBCN. The positive relationship between Soil C and fungal diversity suggests that greater carbon availability may increase substrate availability and create more favorable conditions for maintaining diverse fungal taxa. This is consistent with the view that soil carbon inputs are important drivers of fungal community diversity by regulating substrate supply and habitat heterogeneity (Chen et al., 2021b). The negative association between MBCN and fungal diversity may indicate that increased microbial biomass does not necessarily correspond to higher fungal diversity. Higher microbial biomass may reflect shifts in resource allocation and microbial community structure, which could influence the relative abundance of fungal taxa and consequently affect diversity patterns (Wang and Kuzyakov, 2024). However, this relationship should be interpreted as an association between microbial biomass and fungal diversity rather than a direct causal effect.

Overall, the SEM results suggest that grazing-related variation in soil fungal diversity was closely associated with changes in plant inputs, soil resource availability, and microbial biomass. This pattern suggests that aboveground vegetation and belowground environmental conditions are important components of the relationships between grazing intensity and fungal diversity, emphasizing the need to consider multiple biotic and environmental pathways when evaluating microbial responses to grassland management.

## 5 Conclusions

Soil fungal communities exhibited depth-dependent responses to grazing intensity, highlighting the importance of considering vertical heterogeneity when evaluating grazing impacts on belowground microbial communities. Fungal community composition was primarily characterized by the prevalence of Ascomycota and Mortierellomycota across grazing treatments and soil layers, while the relative abundance of individual fungal groups varied with grazing intensity and soil conditions. Grazing effects on fungal richness differed among soil layers, with light and moderate grazing generally maintaining higher fungal richness in the surface soil than heavy grazing, whereas fungal responses in deeper soil layers showed distinct patterns associated with differences in resource availability and environmental conditions. Co-occurrence network analysis revealed variation in fungal network organization among grazing intensities. Moderate grazing was associated with higher network complexity and connectivity, whereas heavy grazing was associated with higher modularity and different connectivity patterns. These patterns suggest that potential associations among fungal taxa may vary with grazing-related differences in environmental conditions and resource availability. Piecewise structural equation modeling revealed that grazing intensity was associated with fungal diversity through interconnected pathways involving plant biomass, soil resources, soil pH, and microbial biomass. Specifically, grazing-induced changes in vegetation inputs and soil properties were linked to changes in microbial biomass and fungal diversity, highlighting the importance of plant-soil interactions in understanding fungal community responses to grazing disturbance.

Overall, this study shows that variation in soil fungal communities across grazing intensities was closely associated with vegetation conditions, soil environments, and microbial processes. Moderate grazing was associated with relatively favorable fungal community patterns in this agro-pastoral grassland, particularly in surface-soil richness and co-occurrence network complexity, whereas these patterns were less evident under heavy grazing.

## Data Availability

The original contributions presented in the study are included in the article/Supplementary material, further inquiries can be directed to the corresponding author/s.
