# Supplementary material for: Grazing intensity shapes soil fungal diversity and co-occurrence networks across soil depths
Source: Front Microbiol. 2026 Sep 30;17:1941617. doi: 10.3389/fmicb.2026.1941617 (PMC13662103; doi:10.3389/fmicb.2026.1941617)
Supplement: Supplementary file 1 [file Table_1.docx]

Supplementary material

**Figure S1.** Effects of grazing intensity and soil depth on soil bulk density. Different uppercase letters indicate significant differences among grazing intensities within the same soil layer, whereas different lowercase letters indicate significant differences among soil layers under the same grazing intensity (*P* < 0.05). CK, ungrazed control; LG, light grazing; MG, moderate grazing; HG, heavy grazing. Grey points represent individual observations from each plot (n = 3 per grazing intensity × soil layer combination).

**Figure S2.** Relative abundance of fungal classes under different grazing intensities and across soil layers. CK, ungrazed control; LG, light grazing; MG, moderate grazing; HG, heavy grazing. The suffixes 1, 2, and 3 represent the 0-2 cm, 2-10 cm, and 10-20 cm soil layers, respectively. Each bar represents the mean relative abundance across three independent plots (n = 3).

**Figure S3.** Relative abundance of fungal orders under different grazing intensities and across soil layers. CK, ungrazed control; LG, light grazing; MG, moderate grazing; HG, heavy grazing. The suffixes 1, 2, and 3 represent the 0-2 cm, 2-10 cm, and 10-20 cm soil layers, respectively. Each bar represents the mean relative abundance across three independent plots (n = 3).

**Figure S4.** Relative abundance of fungal families under different grazing intensities and across soil layers. CK, ungrazed control; LG, light grazing; MG, moderate grazing; HG, heavy grazing. The suffixes 1, 2, and 3 represent the 0-2 cm, 2-10 cm, and 10-20 cm soil layers, respectively. Each bar represents the mean relative abundance across three independent plots (n = 3).

**Figure S5.** Relative abundance of fungal genera under different grazing intensities and across soil layers. CK, ungrazed control; LG, light grazing; MG, moderate grazing; HG, heavy grazing. The suffixes 1, 2, and 3 represent the 0-2 cm, 2-10 cm, and 10-20 cm soil layers, respectively. Each bar represents the mean relative abundance across three independent plots (n = 3).

**Table S1.** Topological properties of fungal co-occurrence networks under different grazing intensities.

| **Grazing intensity** | **Nodes** | **Edges** | **Positive correlation**  **(%)** | **Average degree** | **Modularity** | **Average clustering coefficient** | **Average path length** |
| --- | --- | --- | --- | --- | --- | --- | --- |
| CK | 46 | 167 | 65.87 | 7.261 | 0.416 | 0.514 | 2.701 |
| LG | 56 | 205 | 50.73 | 7.321 | 0.399 | 0.492 | 2.986 |
| MG | 59 | 297 | 58.92 | 10.068 | 0.394 | 0.551 | 2.330 |
| HG | 50 | 187 | 55.61 | 7.480 | 0.440 | 0.571 | 2.695 |

**Table S2.** Principal component analysis (PCA) results for composite variables used in the structural equation model.

| **Parameters** | **Indicators** | **Variance explained by PC1 (%)** |
| --- | --- | --- |
| **Soil C** | SOC, DOC | 53.51 |
| **MBCN** | MBC, MBN | 93.75 |
| **AGBLB** | AGB, LB | 91.43 |
| **FD** | ACE, Chao1, Shannon | 77.22 |

Abbreviations: SOC, soil organic carbon; DOC, dissolved organic carbon; NH_4_^+^-N, ammonium nitrogen; NO_3_^-^-N, nitrate nitrogen; MBC, microbial biomass carbon; MBN, microbial biomass nitrogen; AGB, aboveground biomass; LB, litter biomass; Soil C, composite variable derived from SOC and DOC; MBCN, composite variable derived from MBC and MBN; AGBLB, composite variable derived from aboveground biomass and litter biomass; FD, fungal diversity; PC1, the first principal component. Soil N represents total inorganic nitrogen and was calculated as the sum of NH_4_^+^-N and NO_3_^-^-N; therefore, it was not included as a PCA-derived composite variable.

**Figure S6.** Standardized indirect and total effects among variables included in the final piecewise structural equation model. The left panel shows standardized indirect effects, and the right panel shows standardized total effects estimated from standardized path coefficients of the final piecewise SEM. Positive and negative values indicate positive and negative standardized effects, respectively. GI, grazing intensity; AGBLB, aboveground biomass and litter biomass; Soil C, soil carbon; Soil N, total inorganic nitrogen; AP, available phosphorus; Soil pH, soil pH; MBCN, microbial biomass carbon and nitrogen; FD, fungal diversity.

**Table S3.** Summary of linear mixed-effects model results for the effects of grazing intensity, soil depth, and their interaction on soil properties, microbial biomass, and fungal α-diversity.

| **Variable** | **Effect** | **NumDF** | **DenDF** | **F value** | **P value** |
| --- | --- | --- | --- | --- | --- |
| **DOC** | Grazing intensity | 3 | 8 | 5.747 | 0.021 |
|  | Soil depth | 2 | 16 | 0.337 | 0.719 |
|  | Grazing × soil depth | 6 | 16 | 1.701 | 0.185 |
| **SOC** | Grazing intensity | 3 | 8 | 0.510 | 0.686 |
|  | Soil depth | 2 | 16 | 253.594 | <0.001 |
|  | Grazing × soil depth | 6 | 16 | 0.723 | 0.637 |
| **NH_4_^+^-N** | Grazing intensity | 3 | 24 | 25.328 | <0.001 |
|  | Soil depth | 2 | 24 | 96.831 | <0.001 |
|  | Grazing × soil depth | 6 | 24 | 27.584 | <0.001 |
| **NO_3_^-^-N** | Grazing intensity | 3 | 8 | 22.269 | <0.001 |
|  | Soil depth | 2 | 16 | 32.649 | <0.001 |
|  | Grazing × soil depth | 6 | 16 | 22.380 | <0.001 |
| **TC** | Grazing intensity | 3 | 8 | 0.530 | 0.674 |
|  | Soil depth | 2 | 16 | 152.354 | <0.001 |
|  | Grazing × soil depth | 6 | 16 | 0.424 | 0.853 |
| **TN** | Grazing intensity | 3 | 8 | 1.764 | 0.232 |
|  | Soil depth | 2 | 16 | 203.526 | <0.001 |
|  | Grazing × soil depth | 6 | 16 | 1.250 | 0.333 |
| **C:N** | Grazing intensity | 3 | 8 | 6.122 | 0.018 |
|  | Soil depth | 2 | 16 | 51.785 | <0.001 |
|  | Grazing × soil depth | 6 | 16 | 3.141 | 0.031 |
| **AP** | Grazing intensity | 3 | 24 | 2.187 | 0.116 |
|  | Soil depth | 2 | 24 | 33.105 | <0.001 |
|  | Grazing × soil depth | 6 | 24 | 2.813 | 0.032 |
| **pH** | Grazing intensity | 3 | 8 | 9.580 | 0.005 |
|  | Soil depth | 2 | 16 | 198.396 | <0.001 |
|  | Grazing × soil depth | 6 | 16 | 13.111 | <0.001 |
| **MBC** | Grazing intensity | 3 | 8 | 0.110 | 0.952 |
|  | Soil depth | 2 | 16 | 124.121 | <0.001 |
|  | Grazing × soil depth | 6 | 16 | 0.653 | 0.688 |
| **MBN** | Grazing intensity | 3 | 8 | 0.687 | 0.585 |
|  | Soil depth | 2 | 16 | 45.026 | <0.001 |
|  | Grazing × soil depth | 6 | 16 | 1.003 | 0.457 |
| **ACE** | Grazing intensity | 3 | 8 | 2.493 | 0.134 |
|  | Soil depth | 2 | 16 | 107.409 | <0.001 |
|  | Grazing × soil depth | 6 | 16 | 2.869 | 0.043 |
| **Chao1** | Grazing intensity | 3 | 8 | 2.586 | 0.126 |
|  | Soil depth | 2 | 16 | 100.847 | <0.001 |
|  | Grazing × soil depth | 6 | 16 | 2.876 | 0.043 |
| **Shannon** | Grazing intensity | 3 | 8 | 0.764 | 0.545 |
|  | Soil depth | 2 | 16 | 2.009 | 0.167 |
|  | Grazing × soil depth | 6 | 16 | 2.039 | 0.119 |
| **Bulk density** | Grazing intensity | 3 | 8 | 1.304 | 0.338 |
|  | Soil depth | 1 | 8 | 68.174 | <0.001 |
|  | Grazing × soil depth | 3 | 8 | 0.296 | 0.827 |

**Note:** NumDF, numerator degrees of freedom; DenDF, denominator degrees of freedom. Linear mixed-effects models were used with grazing intensity and soil depth as fixed effects and plot as a random effect. *P* < 0.05 was considered statistically significant.

Supplementary R scripts used for the linear mixed-effects models, PERMANOVA, co-occurrence network analysis, and piecewise structural equation modeling are provided as separate supplementary files.
